# Kissing the Book

**Published:** 1903-07-11

**Authors:** 


### Editor's Letter-Box.

**[Our Correspondents are reminded that prolixity is a great bar to publication, and that brevity of style and conciseness of statement greatly facilitate early insertion.]**

"KISSING THE BOOK."

Mr. F. R Humphreys writes: "Sir, May I point out that under the provisions of 'An Act to Amend the Law as to Oaths,' A.D. 1888, 51 & 52 Vict., Clause 5, any person desiring to take an oath in the Scottish fashion '*shall* be permitted to do so, and the oath *shall* be administered to him in such form and manner without further question.' The last part of this quotation is perhaps as important as the first, though little difficulty will be experienced. A copy of the Act, price ½d., may be obtained through any bookseller."

